# Cholinergic Stimulation by Pyridostigmine Bromide Before Myocardial Infarction Prevent Cardiac and Autonomic Dysfunction

**DOI:** 10.1038/s41598-019-38841-y

**Published:** 2019-02-21

**Authors:** C. A. Barboza, A. R. Fukushima, N. Carrozzi, J. F. Machi, P. M. M. Dourado, C. T. Mostarda, M. C. Irigoyen, L. Nathanson, M. Morris, E. C. Caperuto, B. Rodrigues

**Affiliations:** 1 School of Physical Education, University of Campinas - UNICAMP, Campinas, SP Brazil; 2 Department of Pathology, School of Veterinary Medicine and Animal Science, University of São Paulo – USP, São Paulo, SP Brazil; 3 Human Movement Laboratory, São Judas Tadeu University - USJT, São Paulo, SP Brazil; 4 Institute of Neuro-Immune Medicine, Nova Southeastern University, Miami, FL USA; 5 Hypertension Unit, Heart Institute (InCor), Medical School of University of São Paulo, São Paulo, SP Brazil; 6 Department of Physical Education, Federal University of Maranhão - UFMA, São Luís, MA Brazil

## Abstract

Inflammatory processes and cardiovascular autonomic imbalance are very relevant characteristic of the enormous dynamic process that is a myocardial infarction (MI). In this sense, some studies are investigating pharmacological therapies using acetylcholinesterase inhibitors, such as pyridostigmine bromide (PYR), aiming to increase parasympathetic tone after MI. Here we hypothesized that the use of PYR before the MI might bring an additional positive effect to the autonomic function, and consequently, in the inflammatory response and cardiac function. The present study aimed to evaluate left ventricular function, baroreflex sensitivity, autonomic modulation, and inflammatory profile in PYR-treated rats previously to MI. **Methods:** Male Wistar rats (250–300 g) were treated for 60 days with PYR. After treatment, they were submitted to the MI. After the MI, the autonomic and ventricular function were evaluated, as well as the systemic, left ventricle, and adipose tissue inflammatory profile. **Results:** PYR, performed before MI, prevented HR increase, systolic function impairment, baroreflex sensitivity drop, as well as pulse interval variance, RMSSD, blood pressure and parasympathetic modulation reduction in treated rats compared to untreated rats. Also, this positive functional changes may have been a result of the reduced inflammatory parameters in the left ventricle (IFN-γ, IL-6, and IL-1β), as well as increased IL-10 expression and IL-10/TNF-α ratio in treated animals before MI. **Conclusion:** Prior treatment with PYR prevents impairment of the autonomic nervous system after MI, which may be associated with the attenuated expression of inflammatory factors and heart dysfunction.

## Introduction

Cardiovascular diseases (CVD) are the significant causes of mortality worldwide, and, among them, myocardial infarction (MI) is the one with the highest mortality rate^1^. In the United States, it is estimated that every 44 seconds an individual has an MI, and about 49% of deaths from cardiovascular disease are attributed to cardiac ischemic events^1^. The knowledge about the clinical characteristics of MI includes: left ventricular dysfunction with loss of cardiomyocytes which results in a decrease of ejection fraction; an increase of residual volume in the left ventricle (LV) and dilatation; as well as the production of inflammatory cytokines by activated fibroblasts, monocytes/macrophages and cardiomyocytes, demonstrating an essential link in the direct and indirect interaction between these cell types. Together, these changes induce a robust pathological cardiac remodeling. Furthermore, hemodynamic damage, by an increase in filling pressures is responsible for pulmonary congestion^2–6^.

The chronic autonomic imbalance between sympathetic and parasympathetic autonomic function^6^, and baroreflex impairment, with greater activation of ergoreflex and chemoreflex, are other factors that must be taken into consideration^7^. Reduction of the parasympathetic tonus, baroreflex and the heart rate variability (HRV) impairment are recognized as a significant predictor of mortality after the MI^6–8^. Thus, alterations caused by cardiovascular autonomic dysfunction can be prevented, especially with increased vagal activity, and may be an essential strategy to control this clinical condition.

Regarding this issue, studies have strengthened the hypothesis of a direct relationship between parasympathetic activation, mediated primarily by the vagus nerve, and immune system response. Thus, the existence of an anti-inflammatory reflex was postulated, whose afferent nerve signaling is activated by cytokines or pathogen-derived products. This afferent signaling is functionally associated with greater efferent activation of the vagus nerve and, consequently, greater release of acetylcholine in the synaptic cleft, negatively regulating the production of proinflammatory cytokines^9–11^. Thus, it is possible to suggest that the reduction of the parasympathetic regulation after the ischemic event could neglect the anti-inflammatory reflex, reducing the action of the cholinergic pathway^5^.

In the search for therapies that improve cardiovascular autonomic balance in favor of the increase of the parasympathetic nervous system, and consequently inflammatory process, our group and other researchers have demonstrated the positive effects of cholinergic stimulation by pyridostigmine bromide (PYR, an cholinesterase inhibitor)^12–15^ and exercise training - known as an crucial vagal modulator^16–18^, as well as the combination of both strategies^19,20^. However, little is known whether indirect vagal stimulation by PYR could prevent the deleterious changes caused by MI. To our knowledge, this is the first study that hypothesized that PYR treatment before MI might protective to the heart. Thus, the present study aimed to evaluate the ventricular, autonomic and inflammatory responses of myocardial ischemia in rats previously treated with PYR.

## Methods

All experiments were approved by the Institution Animal Care and Use Committee of the São Judas Tadeu University (CEUA/USJT - 008/2013) and the study was conducted in accordance with the Guide for the care and Use of Laboratory Animals, issued by the National Institutes of Health (NIH Publication number 96–23, revised in 1996). Male Wistar rats (250–300 g) were obtained from the Animal House of the São Judas Tadeu University, São Paulo, Brazil. Rats were fed standard laboratory chow and water *ad libitum* and were housed in collective polycarbonate cages, in a temperature-controlled room (22 °C) under a 12 h dark–light cycle (lights on 07:00–19:00 hours). The animals were randomly assigned into three groups: MI (I, n = 13), infarcted animals treated with PYR (TPI, n = 10), and control (C, n = 9). TPI was treated with PYR (Sigma^®^, St Louis, MO, USA) dissolved in water (0.14 mg/mL water) available *ad libitum*, as described previously^14,15,19,20^. PYR solutions were prepared daily, and the water bottles were wrapped with a black paper. PYR treatment started immediately at the begin of the protocol and occurred continuously for 8 weeks until MI surgery. Water consumption and the mortality rate were monitored during the experimental period in the PYR-treated and untreated groups. The potential influence of surgical and anesthetic procedures was excluded from this analysis.

### Experimental Design

The experimental protocol was carried out over a total of 9 weeks. The first eight weeks consisted of PYR treatment, or only water, before MI (i.e., TPI and I, respectively), while animals allocated in the C group were submitted to sham surgery. During the following week, the animals were subjected to echocardiography analysis (first-day post-surgery), catheterization of the femoral artery and vein for hemodynamic assessment (second-day post-surgery), baroreflex sensitivity and autonomic tone evaluations (third-day post-surgery). One day after the last assessment, animals were killed by decapitation to remove the left ventricle (LV), periepididymal adipose tissue, and blood (i.e., plasma) for inflammatory profile analysis^19,20^.

### Myocardial Infarction Surgery

Anesthetized rats (80 mg/kg ketamine and 12 mg/kg xylazine, i.p.) underwent surgical occlusion of the left coronary artery, which resulted in MI as described previously^15,17–20^. Briefly, after intubation, animals were positive-pressure ventilated with room air at 2.5 mL, 65 strokes/min with a pressure-cycled rodent ventilator (Harvard Apparatus, Model 683, Holliston, MA, USA). For induction of MI, a 2-cm left lateral thoracotomy was performed in the third intercostal space, and the left anterior descending coronary artery was occluded with nylon (6.0) suture at approximately 1 mm from its origin below the tip of the left atrium. The C animals underwent the same procedures except for myocardial ischemia, which was not induced (Sham surgery). The chest was closed with a silk suture.

### Transthoracic Echocardiography

Echocardiographic evaluations were performed, by a blinded observer, to obtain MI akinetic area, systolic and diastolic parameters. These analyses were realized under the guidelines of the American Society of Echocardiography. Rats were anesthetized (80 mg/kg ketamine and 12 mg/kg xylazine, i.p.) and images were obtained with a 10–14 MHz linear transducer in a SEQUOIA 512 (ACUSON Corporation, Mountain View, CA, USA) for measurements of left ventricular mass (LVMass); left ventricular end-diameter during diastole (LVDD); relative wall thickness (RWT); ejection fraction (EF), left ventricular fractional shortening (LVFS), E wave A wave ratio (E/A ratio); myocardial performance index (MPI). Through midtransversal and apical transversal views, MI size was measured by bi-dimensional echocardiogram. In diastole, three measurements of the endocardial perimeter (EP) and the length of the infarcted segment (ISe) were obtained for each view. MI size for each segment (ISi) was calculated by the equation ISi (%) = ISe/EP × 100. The estimation of the infarct size of each animal was calculated as the mean of ISi (%) of the three segments. MI size was defined as increased echogenicity and/or change in myocardial thickening or systolic movement (hypokinesia, akinesia, or dyskinesia). Our group and other researchers have previously shown strong correlations between the MI area assessed by echocardiogram and post-mortem histological analysis^19,20^.

### Hemodynamic, Baroreflex Sensitivity and Autonomic Tonus Measurements

One day after echocardiographic evaluations, two catheters filled with 0.6 mL heparinized saline solution (the solution was prepared with 0.9 mL of saline and 0.1 mL of heparin) and were implanted into the carotid artery and jugular vein. The catheters were exteriorized on the back of the neck, between the scapulae of the rat, while the animals were anesthetized (80 mg/kg ketamine and 12 mg/kg xylazine, i.p.) for direct measurements of arterial pressure (AP) and vasoactive drug administration, respectively. Rats were studied one day after catheter placement; they were conscious and allowed to move freely during the experiments. The catheters were flushed with a 0.6 mL heparinized saline solution before the experiments. The arterial cannula was connected to a strain-gauge transducer (Blood Pressure XDCR; Kent Scientific, Torrington, CT, USA), and AP signals were recorded over 30 minutes by a microcomputer equipped with an analog-to-digital converter board (WinDaq, 2 kHz, DATAQ, Springfield, OH, USA). The recorded data were analyzed on a beat-to-beat basis to quantify changes in mean AP and heart rate (HR)^15,17–20^.

A sequential bolus injection of increasing doses of phenylephrine (PHE: 0.25 to 32 μg/kg) and sodium nitroprusside (SNP: 0.05 to 1.6 μg/kg) were given to induce at least four pressure responses (for each drug) ranging from 5 to 40 mmHg. A 3–5 min interval between doses was necessary for AP to return to baseline. Peak increases or decreases in mean AP after PHE or SNP injection and the corresponding peak reflex changes in HR were recorded for each dose of the drug. Baroreflex sensitivity (BrS) was evaluated by a mean index, calculated by the ratio between changes in HR and changes in mean AP, allowing a separate analysis of bradycardic (BR) and tachycardic responses (TR). The mean index was expressed as bpm/mmHg, according to previous studies^15,17–20^.

Vagal (VT) and sympathetic tone (ST), as well as intrinsic heart rate (IHR), were measured by determining the response to methylatropine (1 mg/kg i.v.) and propranolol (1 mg/kg i.v.) with a maximal injection volume of 40 μl in a 2-day protocol. Because the heart rate (HR) responses to methylatropine and propranolol reach their peak in 3 to 5 minutes, this time interval was used to quantify the drug-induced HR changes. On the first day of study, resting HR was recorded while the rats were in their home cages in an unrestrained state. After methylatropine injection, AP and HR were recorded for 5 min. Propranolol was injected 6 min after methylatropine, and the response was measured for 5 min. The IHR was evaluated after the combined treatment with propranolol and methylatropine. On the second day, the sequence of injections was inverted, beginning with the propranolol injection. The methylatropine effect was evaluated as the difference between the maximum HR after methylatropine and the control HR. The propranolol effect was evaluated as the difference between the control HR and minimum HR produced after propranolol injection^15^.

### Cardiovascular Autonomic Modulation

The variance of the time series assessed the overall variability of the systolic AP (SAP) in the time domain. Fluctuations in SAP were evaluated in the frequency domain using autoregressive spectral estimation. The theoretical and analytical procedures for autoregressive modeling of oscillatory components have been previously described^17–21^. Briefly, the SAP series, derived from each recording, was divided into 300 beat segments with a 50% overlap. The spectra of each segment were calculated via the Levinson-Durbin recursion and the order of the model chosen according to Akaike’s criterion, with the oscillatory components quantified in low frequency (LF; 0.2–0.6 Hz) and high-frequency (HF; 0.6–3.0 Hz) ranges^21^. One day after *in vivo* measurements, the animals were killed by decapitation and blood, left ventricular and periepididymal adipose tissue tissues were removed. The intact tissue of the left ventricle was dissected, separated and stored to evaluate the inflammatory profile.

### Acetylcholinesterase Activity

Blood samples were collected in vials containing 30 μl of EDTA (0.1 M, Sigma-Aldrich). After centrifugation (3,000 g; 4 °C) for 20 min, plasma was collected and kept at −20 °C until the determination of acetylcholinesterase (AChE) activity. Enzymatic assays were performed using an adaptation of the colorimetric method, as described by Lataro *et al*.^22^ and previously used by our group^19,20^. Plasma samples (10 μl) were incubated in 96-well microplates with 0.01 M 5,5′-dithio-bis-(2-nitrobenzoic acid, DTNB, Sigma-Aldrich) and the excess of the substrate (acetylthiocholine, 0.075 M, Sigma-Aldrich) in 0.2 mM phosphate buffer, pH 8.0 at 30 °C, in the presence of the selective inhibitor of butyrylcholinesterase, tetraisopropylpyrophosphoramide (IsoOMPA 10-3 M, Sigma-Aldrich). Generation of the reaction product was followed in a microplate reader (BioTek FL600, BioTek Instruments, Winooski, VT) at 405 nm for 60 min at 3-min intervals. The maximum velocity (Vmax) of the reaction for each sample was determined in duplicate and expressed as units per liter of plasma (U/L).

### Cytokines Concentration

Serum, LV muscle (intact tissue) and periepididymal adipose tissue levels of proinflammatory cytokines (PICs) (tumor necrosis factor alpha [TNF-𝛼], interferon gama [IFN-γ], interleukin-1 beta [IL-1β], interleukin-6 [IL-6]), and anti-inflammatory cytokines (interleukin-10 [IL-10]) were determined by ELISA (DuoSet ELISA, R&D Systems, Minneapolis, MN, USA) in accordance with the manufacturer’s instructions. All samples were run as duplicates, the mean value was reported, and the results were normalized by LV total protein extracted by the Bradford method^17–20^.

### Statistical Analysis

Statistical calculations were performed using SPSS version 20.0 (Chicago, IL, USA). Data reported as a mean ± standard error of the mean. After confirmed that all variables were normally distributed using the Kolmogorov-Smirnov test, statistical differences between the groups were obtained using two-way analysis of variance (ANOVA) followed by Bonferroni posthoc test to compare the groups. All tests were two-sided, and the significance level was established at *P* < 0.05.

## Results

### Mortality

No deaths were registered during the 8-week treatment protocol in any of the groups. After MI, one animal died in the I group, while no deaths were observed in C and TPI. Therefore, the final sample sizes for C, I, and TPI consisted of 9, 12, and 10 animals, respectively.

### Body Weight, Water Intake and AChE Activity

Initial body weights were similar between the groups (mean = ~275 g). At the end of the protocol, body weights were increased in the experimental groups when compared to their initial assessment (C = 422 ± 5 g; I = 410 ± 5 g, and TPI = 374 ± 25 g). Water consumption was similar between the experimental groups (C = 56 ± 3 mL/day; I = 61 ± 2 mL/day; TPI = 58 ± 7 mL/day). The mean PYR ingestion in TPI was of 25.6 ± 3.1 mg/kg. AChE activity was measured to confirm the effectiveness of PYR treatment. As expected, AChE activity was significantly reduced in the PYR-treated group (TPI = 412.6 ± 25.1 U/L) when compared to untreated groups (C = 933.8 ± 37.8 U/L; and I = 923.5 ± 44.7 U/L).

### Left Ventricular Function

**Figure 1:**
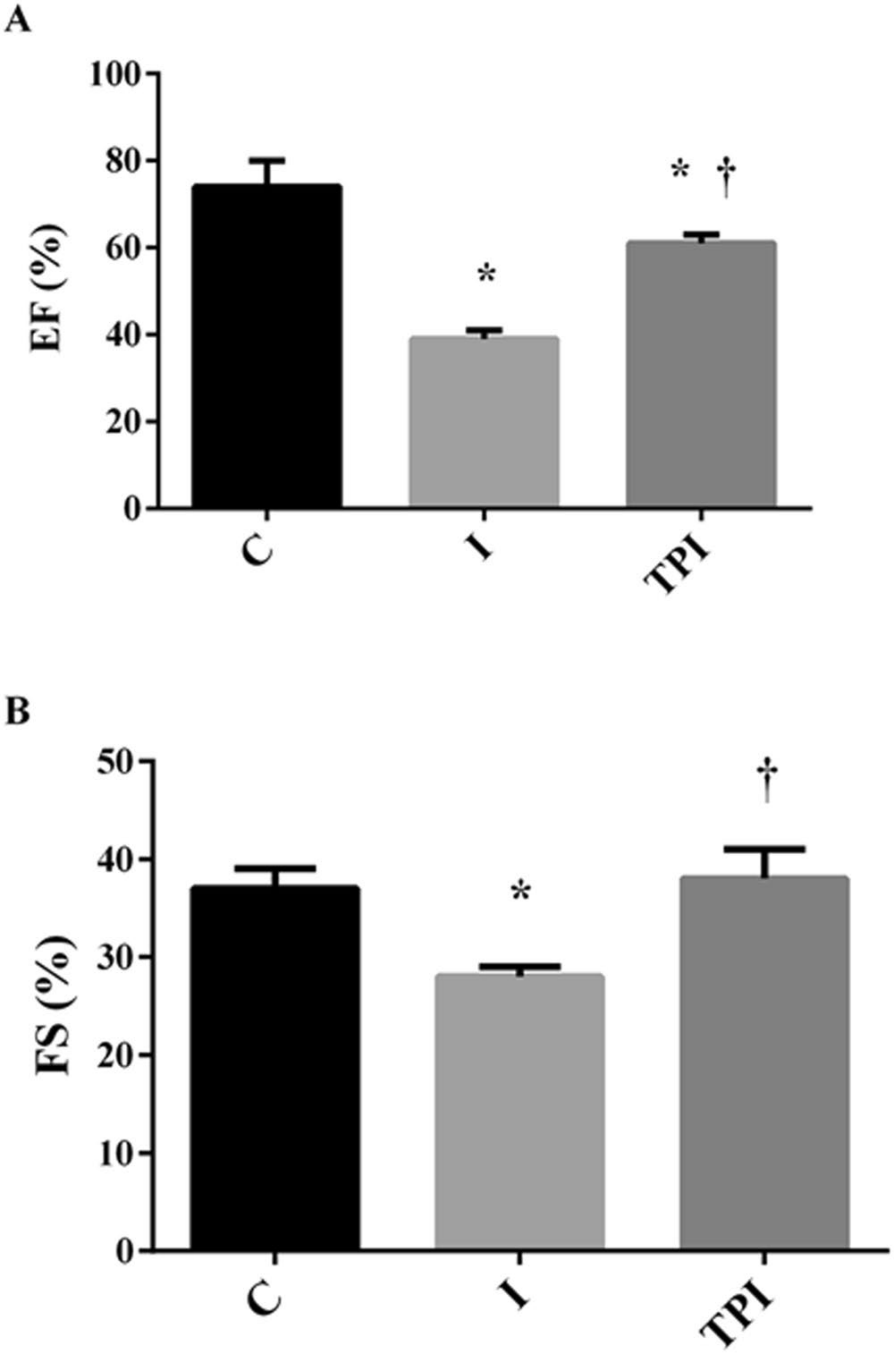
Systolic function evaluated by echocardiogram. Two-way ANOVA with Bonferroni posttest. (**A**) EF – ejection fraction; (**B**) FS – fractional shortening. *P < 0.05 vs. C; ^†^P < 0.05 vs. I.

Table 1 and Fig. 1 show the echocardiographic evaluation in experimental groups. There were no differences on LV mass, LV diastolic diameter, and relative wall thickness among the groups. However, a smaller MI akinetic area was observed in TPI (21 ± 2%) in comparison to I group (46 ± 3%). Also, TPI showed a lower impairment on LV systolic function, assessed trough EF (Fig. 1A) and LVFS (Fig. 1B), when compared to the I group, suggesting a positive impact of PYR treatment in preventing LV systolic dysfunction after MI.

**Table 1:** Echocardiographic measurements.

	C	I	TPI
LVM (g)	1.18 ± 0.03	1.16 ± 0.05	0.97 ± 0.04
LVEDD (cm)	0.68 ± 0.01	0.71 ± 0.03	0.66 ± 0.02
RWT	0.37 ± 0.02	0.35 ± 0.03	0.31 ± 0.04
E/A	1.58 ± 0.15	2.63 ± 0.11*	2.44 ± 0.17*
IVRT (ms)	32 ± 4	34 ± 5	29 ± 2
MPI	0.35 ± 0.02	0.44 ± 0.06	0.46 ± 0.05

Values expressed as mean ± SEM. Two-way ANOVA with Bonferroni posttest. LVM – left ventricle mass; LVEDD – left ventricle end diastolic diameter; RWT – relative wall thickness; E/A - E wave A wave ratio; IVRT – isovolumetric relaxation time; MPI – myocardial performance index. *P < 0.05 vs. C.

### Hemodynamic Measurements

**Figure 2:**
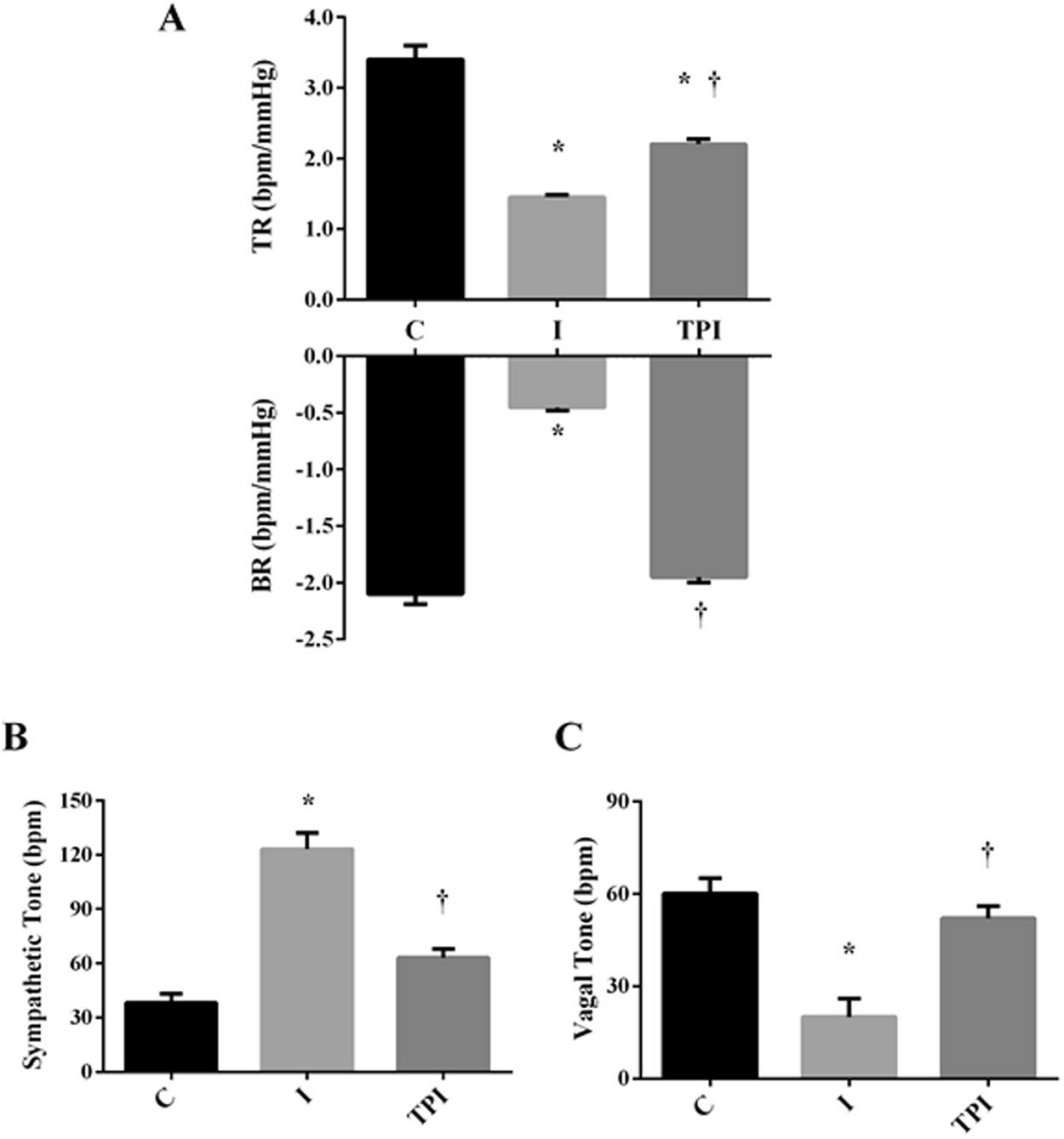
(**A**) Baroreflex sensitivity evaluated by tachycardic and bradycardic responses. (**B**) Sympathetic tone. (**C**) Vagal tone. *P < 0.05 vs. C; ^†^P < 0.05 vs. I.

The values of systolic (SAP), diastolic (DAP), mean (MAP) arterial pressures, and heart rate (HR) were evaluated directly by femoral artery catheterization and are presented in Table 2. There were no differences in SAP, DAP, and MAP among the experimental groups. However, I group showed an increased HR, while PYR treatment in TPI animals prevented this phenomenon. Baroreflex sensitivity, assessed trough tachycardic and bradycardic responses to pharmacological exposure, is shown in Fig. 2A. I group showed a significant impairment in baroreflex sensitivity, while values in TPI group were different from those observed in I group. Moreover, there were no differences between the bradycardic responses observed in C and TPI.

**Table 2:** Hemodynamic parameters.

	C	I	TPI
SAP (mmHg)	119 ± 8	117 ± 9	122 ± 7
DAP (mmHg)	78 ± 5	83 ± 9	81 ± 10
MAP(mmHg)	94 ± 7	89 ± 9	96 ± 12
HR (bpm)	315 ± 12	347 ± 7^*^	288 ± 7^†^

Values expressed as mean ± SEM. Two-way ANOVA with Bonferroni posttest. SAP – systolic arterial pressure; DAP – diastolic arterial pressure; MAP – mean arterial pressure; HR – heart rate. P < 0.05 vs. C; ^†^P < 0.05 vs. I.

### Cardiovascular Autonomic Variables

Autonomic tonus, evaluated by the pharmacological double blockade, evidenced that I group displayed increased values of sympathetic tone (Fig. 2B) and reduced vagal tone (Fig. 2C) about C. However, prior treatment with PYR inhibited changes on sympathetic and vagal tonus on TPI. There were no changes on intrinsic heart rate values in the experimental groups (C = 357 ± 15 bpm; I = 338 ± 10 bpm; TPI = 353 ± 13 bpm).

Pulse interval and systolic arterial pressure variabilities are shown in Table 3. VAR-PI and RMSSD values were reduced in I and TPI groups when compared to the C group. However, values showed by TPI were higher in comparison to I. Regarding frequency domain, was possible to observe that MI caused a substantial autonomic dysfunction, which could be prevented by prior PYR treatment. Indeed, I animals showed a significant reduction in LF and HF spectral bands, as well as on LF/HF ratio when compared to C animals. On the other hand, LF and HF bands were increased in TPI animals in comparison to I, while similar LF/HF ratio was observed between TPI and C.

**Table 3:** Pulse interval and systolic arterial pressure variabilities parameters.

	C	I	TPI
***Pulse Interval Variability***			
Var-IP (ms^2^)	119 ± 15	21 ± 2^*^	64 ± 7^*†^
RMSSD (ms^2^)	6.9 ± 0.2	3.6 ± 0.1^*^	6.2 ± 0.1^†^
LF (ms^2^)	6.2 ± 0.9	0.6 ± 0.1^*^	3.4 ± 0.6^†^
LF (%)	25.3 ± 1.9	13.8 ± 1.1^*^	23.3 ± 2.1^†^
HF (ms^2^)	16.6 ± 1.1	3.8 ± 0.2^*^	13.6 ± 0.9^†^
HF (%)	74.8 ± 2.4	87.2 ± 2.2	76.3 ± 3.0
LF/HF	0.39 ± 0.03	0.17 ± 0.04^*^	0.33 ± 0.06
***Systolic Arterial Pressure Variability***			
Var-SAP (mmHg^2^)	26.7 ± 2	30.1 ± 7	33.4 ± 7
LF (mmHg^2^)	6.5 ± 0.8	7.9 ± 1.3	5.9 ± 0.8
α index (LF, ms/mmHg)	1.32 ± 0.21	0.33 ± 0.05^*^	0.86 ± 0.07

Values expressed as mean ± SEM. Two-way ANOVA with Bonferroni posttest. Var-IP – pulse interval variance; RMSSD - square root of the mean of successive RR interval differences; LF – low-frequency band; HF – high-frequency band; Var-SAP – systolic arterial pressure variance. *P < 0.05 vs. C; ^†^P < 0.05 vs. I.

Regarding SAP modulation, there were no differences in Var-SAP and LF-SAP parameters among the groups. However, spontaneous baroreflex, assessed through the alpha index, was reduced in I group in comparison to the C group. No differences on spontaneous baroreflex were observed between TPI and C group (Table 3).

### Inflammatory Profile

**Figure 3:**
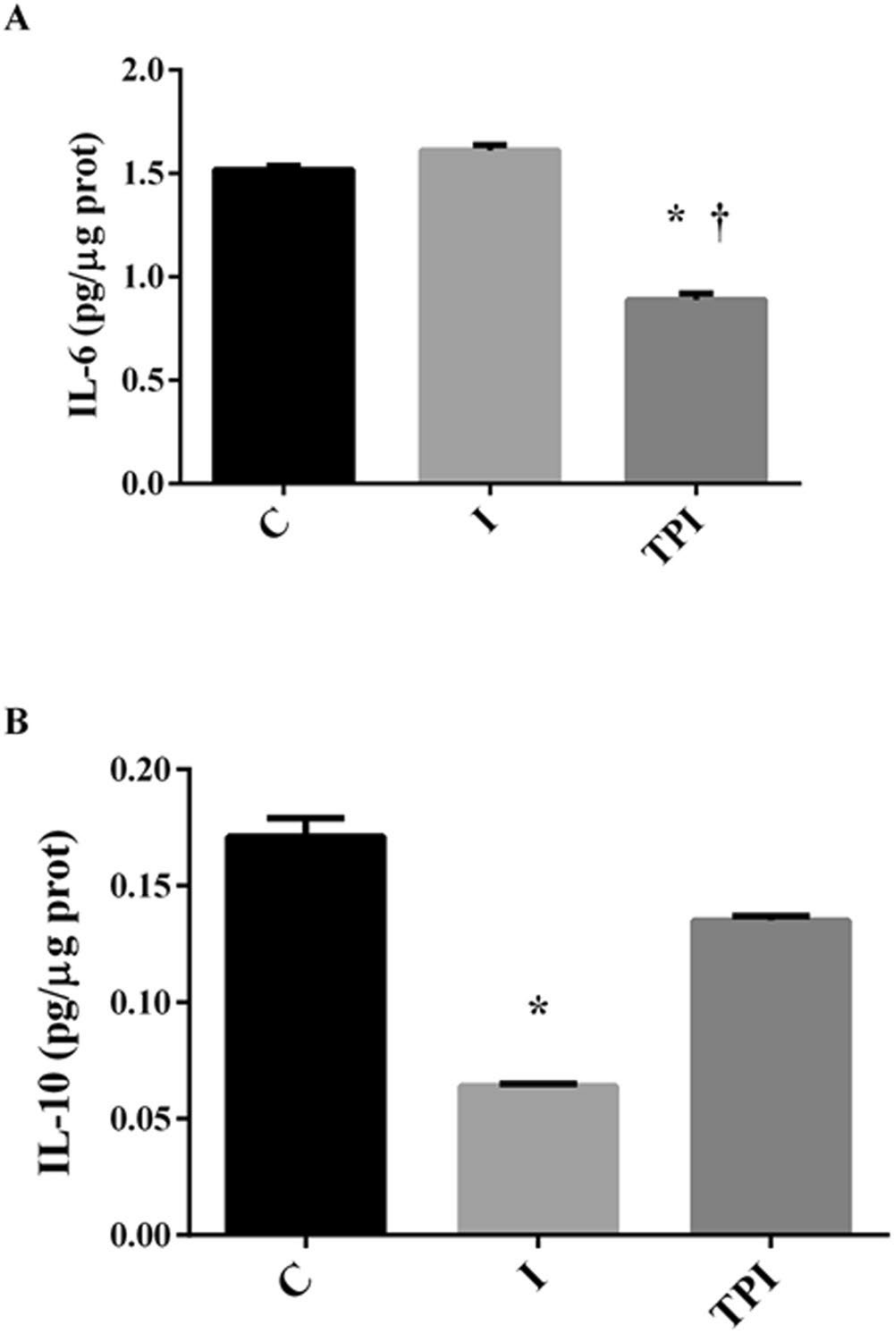
Serum inflammatory markers. (**A**) IL-6. (**B**) IL-10. *P < 0.05 vs. C; ^†^P < 0.05 vs. I.

**Figure 4:**
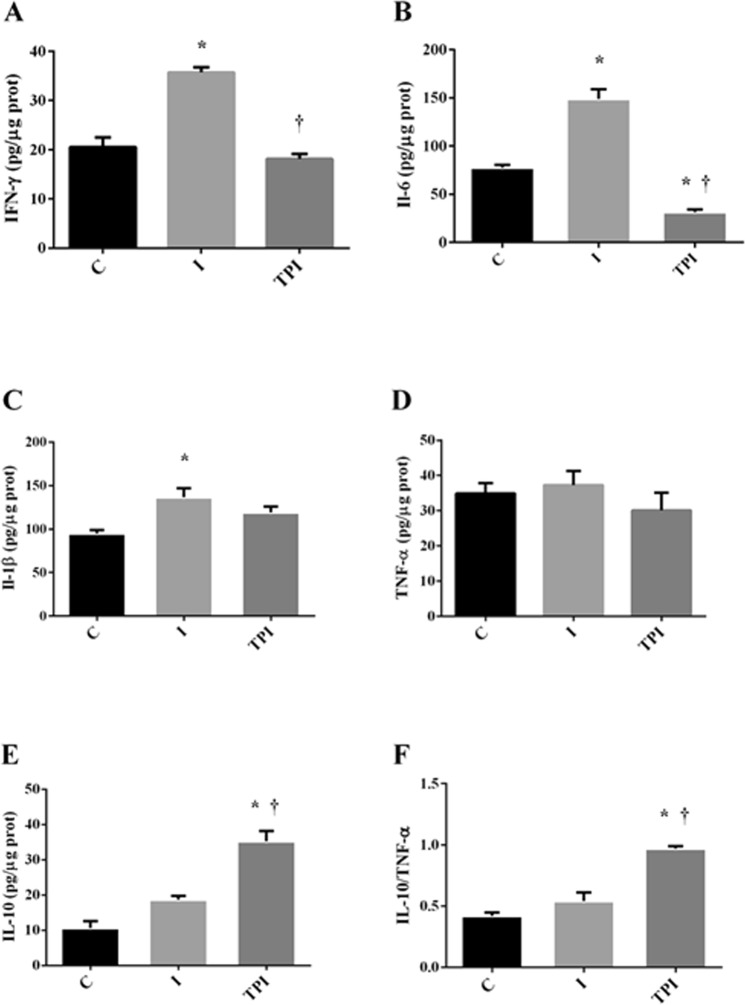
Inflammatory markers on the left ventricle. (**A**) IFN-γ. (**B**) IL-6. (**C**) IL-1β. (**D**) TNF-α. (**E**) IL-10. (**F**) IL-10/ TNF-α. *P < 0.05 vs. C; ^†^P < 0.05 vs. I.

**Figure 5:**
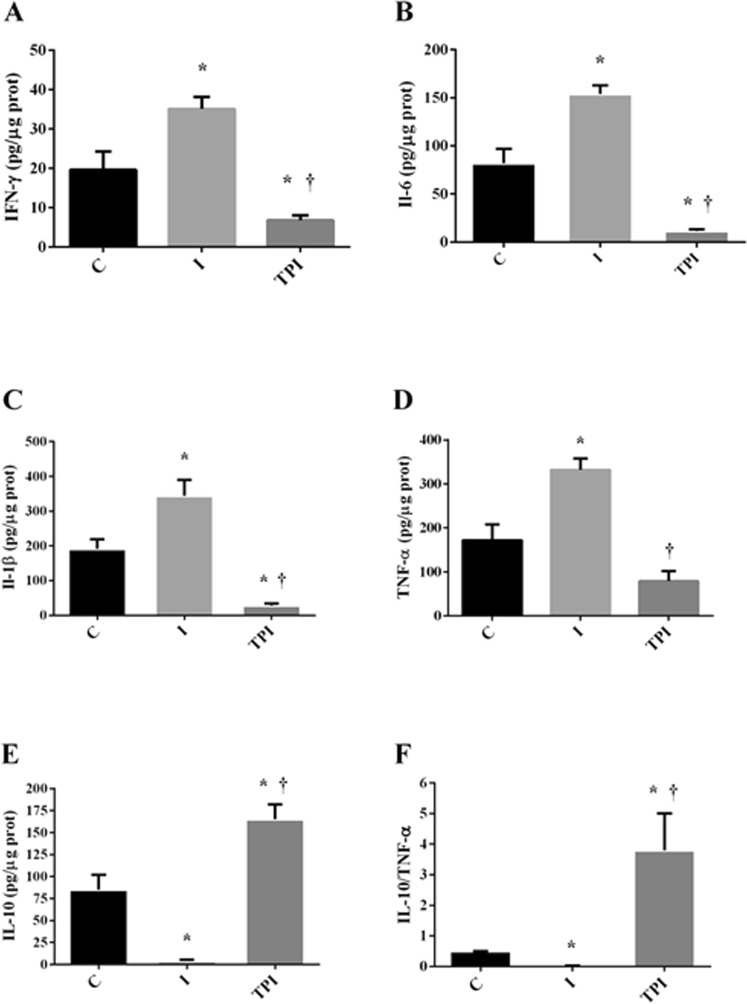
Inflammatory markers on white adipose tissue. (**A**) IFN-γ. (**B**) IL-6. (**C**) IL-1β. (**D**) TNF-α. (**E**) IL-10. (**F**) IL-10/ TNF-α. *P < 0.05 vs. C; ^†^P < 0.05 vs. I.

Inflammatory and anti-inflammatory markers are shown in Figs 3–5.

#### Systemic

I group showed a significant reduction in serum IL-10 levels when compared to C group, while no changes in IL-10 levels were observed in TPI group (Fig. 3B). Also, PYR treatment elicited significant reductions on serum IL-6 levels in comparison to I and C (Fig. 3A).

#### Left Ventricle

IFN-γ (Fig. 4A), IL-6 (Fig. 4B), and IL1-β (Fig. 4C) concentrations were increased in I group when compared to the C group. These effects were prevented by the prior treatment with PYR in TPI. Furthermore, it should be stressed that IL-10 concentration (Fig. 4E) and IL-10/TNF-α (Fig. 4F) ratio were significantly increased in TPI group in comparison to I and C. TNF-α (Fig. 4D) concentration was similar among the groups.

#### Periepididymal Adipose Tissue

I group showed increased IFN-γ (Fig. 5A), IL-6 (Fig. 5B), IL1-β (Fig. 5C), and TNF-α (Fig. 5D) concentrations, as well as lower IL-10 (Fig. 5E) and IL-10/TNF-α ratio (Fig. 5F) concentrations when compared to C group. Prior PYR treatment significantly inhibited all inflammatory changes induced by MI on periepididymal adipose tissue. TPI group showed lower IFN-γ (Fig. 5A), IL-6 (Fig. 5B), IL1-β concentrations in comparison to I, and higher IL-10 (Fig. 5E) and IL-10/TNF-α ratio (Fig. 5F) concentrations when compared to I and C.

## Discussion

The main findings of the present study demonstrated that PYR treatment before MI might prevent heart dysfunction commonly observed after an ischemic cardiac event. According to our results, this preventive phenomenon occurred in response to a positive impact of PYR treatment on systemic and tissue inflammatory profile, as well as on autonomic modulation.

After MI, myocyte necrosis results in a disruption of cardiac metabolism, mainly characterized by inadequate production of creatine phosphate and adenosine triphosphate and accumulation of degradation products, causing the migration of immunological cells (e.g., monocytes, neutrophils) into the infarcted zone^23–25^. Subsequently, matrix metalloproteinases (MMPs) released for neutrophils will cause disintegration and degradation of the interfibrillar collagen. As a result, the cardiac wall of the infarcted area gets thinner, and the ventricular cavity dilates, a phenomenon known as infarct expansion^23–25^. These adjustments induced by MI lead to the loss of myocardium functionality (e.g., reduction in ejection volume) with a consequent perturbation in circulatory hemodynamics^23–25^. At the same time, in attempt to preserve stroke volume and sufficient blood perfusion to the tissues, the sympathetic branch of the autonomic nervous system is invoked and triggers the activation of other auxiliary systems (e.g., renin-angiotensin-aldosterone system)^6,7^.

In the present study, the infarcted rats showed a post-infarction phenotype similar to those above, confirming that the ligation of the left anterior descending coronary artery is a useful model to reproduce human MI in pre-clinical models^26^. Indeed, EF and LVFS were significantly impaired in the 24 hours post-MI surgery, probably caused by 46% of the akinetic area of left ventricle. Concomitantly, various inflammatory markers (i.e., IFN-γ, IL-6, and IL1-β) were highly expressed in the LV and adjacent tissues (i.e., periepididymal adipose tissue). It was also possible to observe an impairment on baroreceptor reflex, combined with a compensatory activity of the autonomic nervous system since the animals showed an elevated sympathetic tone, LF/HF ratio, and HR, while the parasympathetic markers, such as RMSSD and vagal tone, were reduced.

On the other hand, the MI akinetic area was approximately two-fold lower in PYR-treated animals (21%). This phenomenon was accompanied by a wholly preserved LVFS and a less impaired EF in comparison to I group. Although BrS, VAR-PI, and RMSSD were also impaired in TPI, these adjustments were significantly lower than in I group. Moreover, sympathetic tone and LH/HF ratio — markers of the sympathetic branch of the autonomic nervous system — were similar to C group after MI induction. About inflammatory profile, PYR markedly prevented the increase in systemic and inflammatory tissue markers in addition to an improved anti-inflammatory profile in comparison to I and C groups.

A possible explanation to the preventive effects of PYR on heart dysfunction could be that the 8-week treatment could have led to increased bioavailability of ACh during and post-MI collaborating to improved cardiac remodeling and consequently a less impaired heart dysfunction. In this sense, prior therapy with PYR may have affected several pathways associated with the degradation of contractile performance, as has been observed when vagal nerve stimulation (VNS)^27,28^ or PYR treatment^12–15,22^ are performed, just prior or during the ischemic event. Such mechanisms can include reduced cardiac mitochondrial dysfunction, the inflammatory cells influx into the infarct area, and MMPs activity, as well as improved cardiac redox state, increased on M2-macrophages and vascular endothelial growth factor (VEGF) expressions.

Regarding the last factor, for example, Lataro *et al*.^22^ observed an increased VEGF protein expression in the LV of infarcted rats after PYR treatment. In the light of our findings, VEGF could have facilitated angiogenic (e.g., increased in capillary an arteriolar length densities) in the cardiac tissue and increased the coronary reserve before the cardiac event, improving ventricular remodeling and preserving heart function after MI^29^.

Nevertheless, it is important to mention that the beneficial effects of ACh bioavailability on mitochondrial dysfunction and cardiac remodeling were entirely abolished by the administration of atropine^27^, suggesting a dominant effect of muscarinic receptor on PYR effects. Also, TPI demonstrated significant rest bradycardia when compared to I group.

Interestingly, at the presynaptic level, the ACh released from the vagal axon terminals inhibits the release of noradrenaline from the sympathetic axon terminals via the activation of muscarinic receptors^30,31^. This sympathetic-parasympathetic interaction occurs mainly in the electric heart conduction system when the level of sympathetic neural activity become progressively less pronounced as the level of vagal activity increases^32^. Therefore, by modifying the rate of degradation of acetylcholine release into the heart, during and post-MI, PYR enhanced the bioavailability of ACh in the neural junction of the sinus and atrioventricular nodes and increased the activity of muscarinic receptors, suppress the augmented sympathetic drive to the heart (i.e., lower sympathetic tone and LH/HF ratio). While reduced HR, extended the diastolic period and coronary perfusion during and after the cardiac event^22,29^, resulting in augmented oxygen supply to the cardiac tissue, preserving LF/HF ratio, reducing the complications associated with cardiac remodeling, and maintaining heart function (i.e., EF and LVSF).

Furthermore, Mostarda *et al*.^33,34^ observed that the degree of impairment on BrS post-MI was correlated with sympathetic overactivation, LV remodeling, and cardiac function, mainly through a pathway involved in calcium handling^33,34^. On the other hand, we observed an attenuated degradation on BrS in the TPI group. Therefore, as mentioned above, more substantial ACh bioavailability could have collaborated with attenuated BrS disfunction, aiding in autonomic control from moment to moment.

Taken together, these findings suggest that PYR-induced higher availability of ACh during and after MI may directly reduce akinetic area due to the regulation of several pathways (e.g., mitochondrial dysfunction, inflammatory cells influx into the infarct area, angiogenesis) associated with cardiac remodeling, resulting in a partially preserved cardiac functioning. Indirectly, PYR may have influenced heart dysfunction via its effects on afferent and efferent autonomic cardiac branches.

It is worth mentioning, that the reduced expression of inflammatory markers, observed in the cardiovascular and adipose tissue would be related to the increased ACh availability, selectively stimulating alpha-7 nicotinic receptor of the peripheral branch of the cholinergic anti-inflammatory pathway, as described by Tracey^9^. The idea is that inflammatory modulation should occur by efferent activation of the vagal nerve, releasing ACh that stimulates macrophages inhibiting the production of pro-inflammatory cytokines, obtaining a parasympathetic response as a reflex to the regulatory organs of the immune system. Therefore, in addition to vagal stimulation, inhibition of acetylcholinesterase has also been shown to reduce proinflammatory cytokine production as demonstrated in preclinical models of sepsis^10^ and MI^12,13^. However, more studies investigating this pathway in MI are still necessary to a more comprehensible understanding.

According to the findings of the present study, the treatment with PYR would be an alternative to patients at cardiovascular risk. Acute cholinergic stimulation with PYR caused bradycardia and reduced the QT interval, without significant collateral effects in patients with heart failure^35^. In patients with heart failure, the drug significantly increased heart rate recovery within the first minute after maximal exercise^36^, improved the oxygen pulse (systolic volume indicator), as well as the HR^37^.

However, evidence in the literature suggests that prolonged exposure to high-dose PYR (i.e., 28 days) may cause essential side-effects on the neuromuscular apparatus, including reduced tetanic tension^38^. Additionally, a recent study-case described the presence of leukocytoclastic vasculitis in a 91-year-old man who was treated for two weeks with PYR^39^.

Therefore, although our findings support the hypothesis that the treatment with PYR may be an important tool in the maintenance of heart function after MI, mainly via the regulation of autonomic modulation and inflammatory state, future studies should investigate animal models with conditions associated with a high risk of MI, such as hypertension in an attempt to propitiate a better clinical design. Moreover, dose- and time-response relationships, toxicity, and side-effects should be deeply investigated before the performance of clinical trials.

In conclusion, PYR presents a promising future in the clinical treatment prior MI, since it attenuated heart dysfunction, autonomic abnormalities, and inflammatory processes in infarcted rats.
